# Excess Transforming Growth Factor-α Changed the Cell Properties of Corneal Epithelium and Stroma

**DOI:** 10.1167/iovs.61.8.20

**Published:** 2020-07-15

**Authors:** Lingling Zhang, Yong Yuan, Lung-Kun Yeh, Fei Dong, Jianhua Zhang, Yuka Okada, Winston W Y. Kao, Chia-Yang Liu, Yujin Zhang

**Affiliations:** 1 School of Optometry, Indiana University, Bloomington, Indiana, United States; 2 Crawley Vision Research Laboratory, Department of Ophthalmology, College of Medicine, University of Cincinnati, Ohio, United States; 3 Department of Ophthalmology, Chang-Gung Memorial Hospital, Linkou, Taiwan; 4 Chang-Gung University College of Medicine, Taoyuan, Taiwan; 5 Department of Ophthalmology, Wakayama Medical University, School of Medicine, Wakayama, Japan; 6 School of Optometry, University of California, Berkeley, California, United States; 7 Department of Chemistry, Indiana University, Bloomington, Indiana, United States

**Keywords:** corneal epithelium, stroma, hyperplasia, TGF-α, transgenic mouse

## Abstract

**Purpose:**

This study is to investigate the corneal anomaly caused by excess transforming growth factor-α (TGF-α) during mouse development.

**Methods:**

Bitransgenic *Kera^RT^*/*TGF-*α mice, generated via cross-mating *tetO-TGF-*α and *Kera^RT^* mice, were induced to overexpress TGF-α by doxycycline commencing at embryonic day 0 or postnatal day 0 to different developmental stages. Bitransgenic mice with doxycycline induction were defined as *TGF-*α*^ECK^* mice (TGF-α excess expression by corneal keratocytes). Mouse eyes were examined by hematoxylin and eosin staining, immunofluorescent staining and transmission electron microscopy. Protein and RNA from mouse cornea were subjected to western blotting and real-time quantitative polymerase chain reaction.

**Results:**

In *TGF-*α*^ECK^* mice, TGF-α overexpression resulted in corneal opacity. Excess TGF-α initially caused corneal epithelial hyperplasia and subsequent epithelium degeneration as the mouse developed, which was accompanied by gradually diminished K12 expression from the periphery of corneal epithelium and increased K13 expression toward the corneal center. Interestingly, K14 was detected in all layers of corneal epithelium of *TGF-*α*^ECK^* mice, whereas it was limited at basal layer of controls. Transmission electron microscopy showed desmosome loss between corneal epithelial cells of *TGF-*α*^ECK^* mice. In *TGF-*α*^ECK^* mice, keratocan expression was abolished; α-SMA expression was increased while expression of *Col1a1, Col1a2*, and *Col5a1* was diminished. Cell proliferation increased in the corneal epithelium and stroma, but not in the endothelium of *TGF-*α*^ECK^* mice.

**Conclusions:**

Excess TGF-α had detrimental effects on corneal morphogenesis during mouse development in that it changed the cell fate of corneal epithelial cells to assume conjunctival phenotypic expression of K13, and keratocytes to myofibroblast phenotype.

Transforming growth factor (TGF)-α belongs to the epidermal growth factor (EGF) family. It binds to the EGF receptor (EGFR) to elicit various signaling transduction cascades by which TGF-α is associated with multiple cellular behaviors, such as proliferation, differentiation, and migration of cells during embryonic development and maintenance of homeostasis in adult.^1^^,^^2^ Bidirectional interaction between epithelial cells and stromal mesenchymal cells mediated via growth factors like TGF-α play essential roles in morphogenesis during development and tissue repair.^3^^–^^5^

The biological functions of TGF-α during cancer pathogenesis and normal tissue morphogenesis have been intensively investigated by in vitro and in vivo systems.^1^^,^^5^^–^^9^ TGF-α knock-out mice displayed pronounced waviness of whiskers and fur, accompanied by eye abnormalities, including a thinner corneal epithelium. A number of TGF-α knock-out mice were born with partially open eyes which became opaque and inflamed by week 5.^1^ In contrast, ectopic expression of TGF-α in the mouse lens led to corneal opacities, cataracts, and microphthalmia of the transgenic mouse.^10^ A milder phenotype was also reported in a similar mouse line when the expression level of TGF-α was not as high in which the corneal epithelium had the normal five to seven cell layers; however, in most transgenic mice overexpressing TGF-α whose corneal epithelium has reduced cell layers around two layers instead of six to seven layers.^11^

To better elucidate the effects of TGF-α on ocular surface development in a more controlled manner, we developed a doxycycline [Dox] inducible TGF-α over expressing *TGF-*α*^ECK^* mouse line (bitransgenic *Kera^RT^/TGF-*α* *[*tetO-TGF-*α] mice fed Dox chow), which derived from cross-mating tetO-TGF-α transgenic mice^12^ and *Kera^rtTA^* knock-in mice.^13^ Our data indicated that excess TGF-α changed cell fate of corneal epithelial cells and stromal keratocytes. *TGF-*α*^ECK^* mice posed detrimental effects on cornea morphogenesis during mouse development.

## Methods

### Generation and Genotyping of *Kera^RT^/tetO-TGF-*α Bitransgenic Mice

Bitransgenic *Kera^RT^/tetO-TGF-*α* *mice were generated via mating transgenic *tetO-TGF-*α^[12]^* *and *Kera^RT^*knock-in mice.^13^ All mice were housed at the Animal Facility of the Indiana University. Animal care and use conformed to the ARVO Statement for the Use of Animals in Ophthalmic and Vision Research. All animal procedures were approved by the Indiana University Institutional Animal Care and Use Committee.

Transgenic mice were identified by genotyping via polymerase chain reaction (PCR) using oligonucleotide primers specific for each transgene as listed in Supplementary Table S1.

### Administration of Dox Chow to Prepare *TGF-*α*^ECK^* Mice

To induce TGF-α overexpression, the bitransgenic *Kera^RT^/tetO-TGF-*α* *mice were subjected to systemic induction by Dox chow (1 g/kg: Custom Animal Diets, Bangor, PA) for different periods of time. To prepare fetal *TGF-*α*^ECK^* mice at different developmental stages, pregnant dams and nursing mothers were given an intraperitoneal injection of Dox (80 µg/g body weight in PBS, pH7.4; Clontech Laboratories, Mountain View, CA) at embryonic day 0 (E0) or postnatal day 0 (P0), respectively; then continuously fed Dox chow (ad libitum) until euthanized at embryonic days E14.5 and E18.5 and postnatal days P0, P7, P14, P21, and P30, respectively. Control animals were littermates containing single transgene, that is, *Kera^RT^* and *TGF-*α (*TetO-TGF-*α).

### Hematoxylin and Eosin Staining and Immunofluorescent Staining

Enucleated eyes were fixed overnight in 4% paraformaldehyde solution in PBS at 4°C, followed by dehydration and paraffin embedding. Deparaffinized and rehydrated tissue sections (5 µm) were stained with hematoxylin and eosin and examined under a stereomicroscope (EVOS FL Auto, Life Technologies, Carlsbad, CA). For immunofluorescent staining, corneal tissue sections were incubated with the primary and secondary antibodies as listed in Supplementary Table S2. Sections were photographed using a Zeiss microscope equipped with a camera (Axiocam Mrm). For data acquisition, we used the Axiovision 4.6 software (Carl Zeiss, Jena, Germany).

### Western Blotting Analysis

Frozen corneas isolated from experimental mice were homogenized in RIPA buffer (#89900 Thermo Fisher Scientific, Waltham, MA) containing 1× protease inhibitor cocktail (P8340, Sigma, St Louis, MO). Tissue lysates (20 µg) from each sample were separated on a 4% to 20% linear gradient Tris-HCl denaturing polyacrylamide Ready Gel (Bio-Rad Laboratories Inc., Hercules, CA) and transferred to PVDF membranes (Whatman, Maidstone, UK). The membranes were incubated with primary antibody overnight at 4°C and then were probed with horse radish peroxidase-conjugated secondary antibody for an hour at room temperature. The signal was detected using an enhanced chemiluminescence assay (Supersignal West Pico, #34080; Thermo Fisher Scientific); then examined and photographed using a VersaDoc 4000MP imaging system (Bio-Rad Laboratories Inc.). Antibodies used in this study were listed in Supplementary Table S2.

### Real-Time Quantitative PCR (RT-qPCR)

Total RNA (10 µg) was isolated from mouse corneal tissues using Trizol reagent (Invitrogen, Carlsbad, CA), then annealed to random primers and reverse transcribed with avian reverse transcriptase (RT) kits (Promega, Madison, WI), according to the manufacturer's instructions. RT-qPCR was performed using the CFX96 real-time system equipped with a C1000 Thermal Cycler (Bio-Rad Laboratories Inc.). After the initial 3-minute denaturing step at 95°C, 40 subsequent cycles at 95°C lasting 15 seconds, 62°C for 15 seconds, and 72°C for 30 seconds were performed. The cycle threshold values were used to calculate the normalized expression of genes of interest against *Gapdh* using Q-Gene software. Primer pairs were listed in Supplementary Table S1.

### Transmission Electron Microscopy

Mouse cornea samples were fixed in 0.1 mol/L cacodylate buffer (pH 7.4) containing 3% glutaraldehyde and 2% paraformaldehyde for 2 hours at 4°C and then preserved in 0.1 mol/L cacodylate buffer (pH 7.4) containing 0.5% glutaraldehyde at 4°C overnight. After refixation in 1% osmium tetraoxide for 1 hour at 4°C, cornea samples were washed in 0.1 mol/L cacodylate buffer (pH 7.4) for 10 minutes three times, and then dehydrated in a graded ethanol series and embedded in Epon 812 epoxy resin (#14120, Electron Microscopy Sciences, Hatfield, PA). Ultrathin 50-nm sections were stained with uranyl acetate and lead citrate and imaged with a Hitachi 7500 transmission electron microscope equipped with an AMT digital camera.

### Statistical Analysis

Statistical analysis was executed with Excel (Excel, Microsoft, Redmond, WA). Data were expressed as mean ± standard deviation. Paired *t* tests were used to analyze the significance of difference. A *P* value of less than 0.05 was considered statistically significant, and a *P* value of less than 0.01 was considered highly statistically significant. Note that for each experiment, at least three mice per group were used. Each experiment was repeated three times.

## Results

### TGF-α Was Overexpressed in the Corneal Keratocytes of Dox-Induced *Kera^RT^/TGF-*α Mice

**Figure 1.**
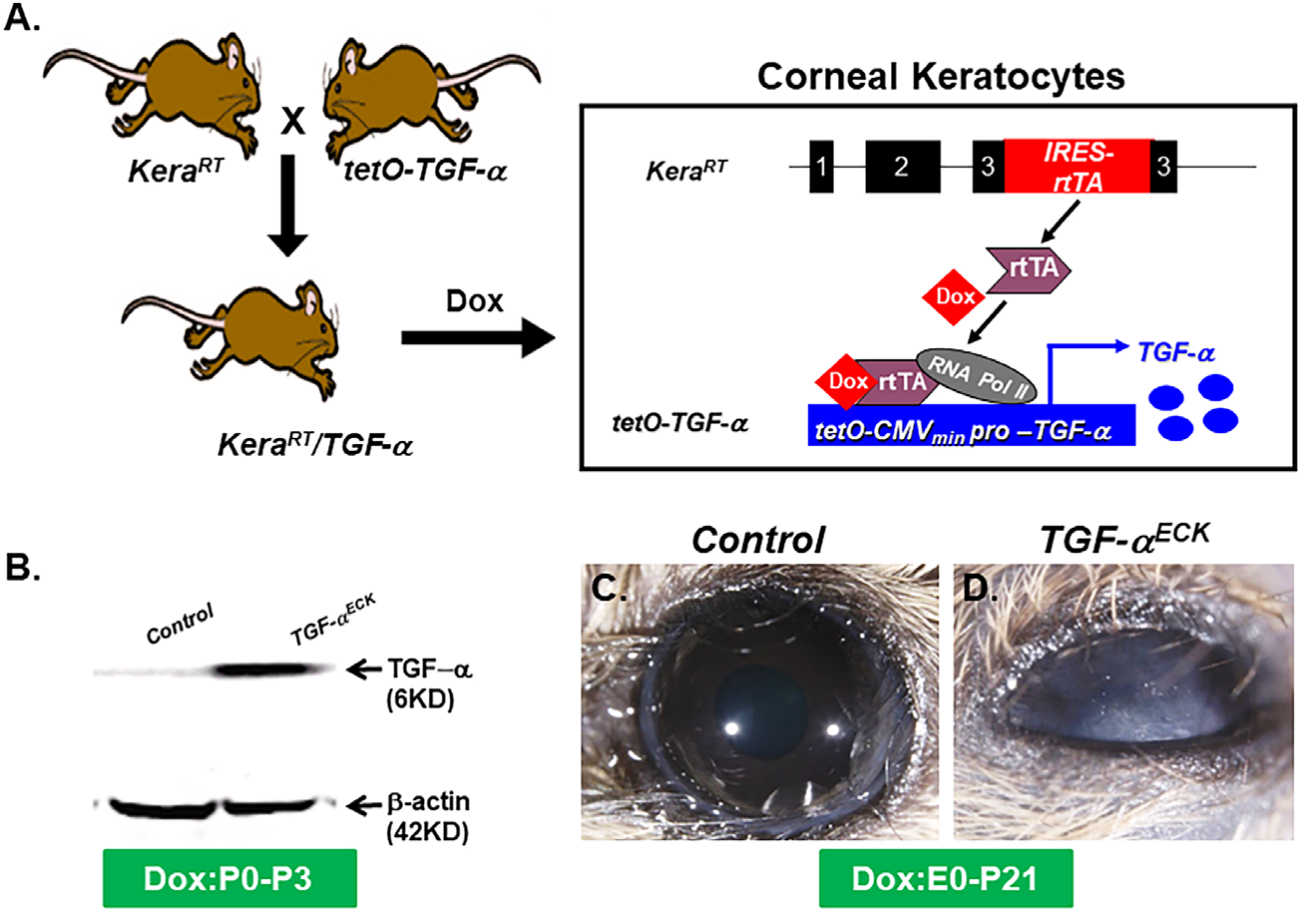
Overexpression of TGF-α in the corneal stroma of *Kera^RT^*/*TGF-*α mice*.* (**A**) Schematic presentation showing *Kera^RT^*/*TGF-*α mice were generated by mating *Kera^RT^* with the *tetO-TGF-*α mouse line. Administration of Dox resulted in overexpression of TGF-α in the keratocytes of corneal stroma. (**B**) Western blot revealed the active form of TGF-α (6 kDa) is dramatically augmented in the corneas of *TGF-*α*^ECK^* (*Kera^RT^*/*TGF-*α mice administered Dox chow from P0 to P3), right lane compared with single transgenic *TGFα* littermate control, left lane. (**C**, **D**) Live images of eyes from double transgenic mice *Kera^RT^*/*TGF-*α and littermate controls at P21 (Dox chow from E0 to P21). Eyes from *Kera^RT^*/*TGF-*α were opaque (**D**), whereas eyes of littermate controls were transparent (**C**).

To conditionally overexpress TGF-α in cornea stromal cells, *Kera^RT^* mice were crossed with transgenic mouse line *tetO-TGF-*α to create the double transgenic mouse *Kera^RT^/TGF-*α. Their single transgenic littermates, *Kera^RT^* or *tetO-TGF-*α, were used as controls (Fig. 1A). First, Western blotting analysis was performed to assess TGF-α expression in cornea of *TGF-*α*^ECK^* mice after Dox induction from P0 until P3. The results revealed that the soluble active TGF-α protein (6 KD) significantly increased in *TGF-*α*^ECK^* mouse cornea (Fig. 1B). Overexpression of TGF-α also caused upregulation of EGFR expression in corneal epithelium and stroma revealed by immunofluorescent staining (Supplementary Fig. S2).

### The Phenotype of *TGF-*α*^ECK^* Transgenic Mice

**Figure 2.**
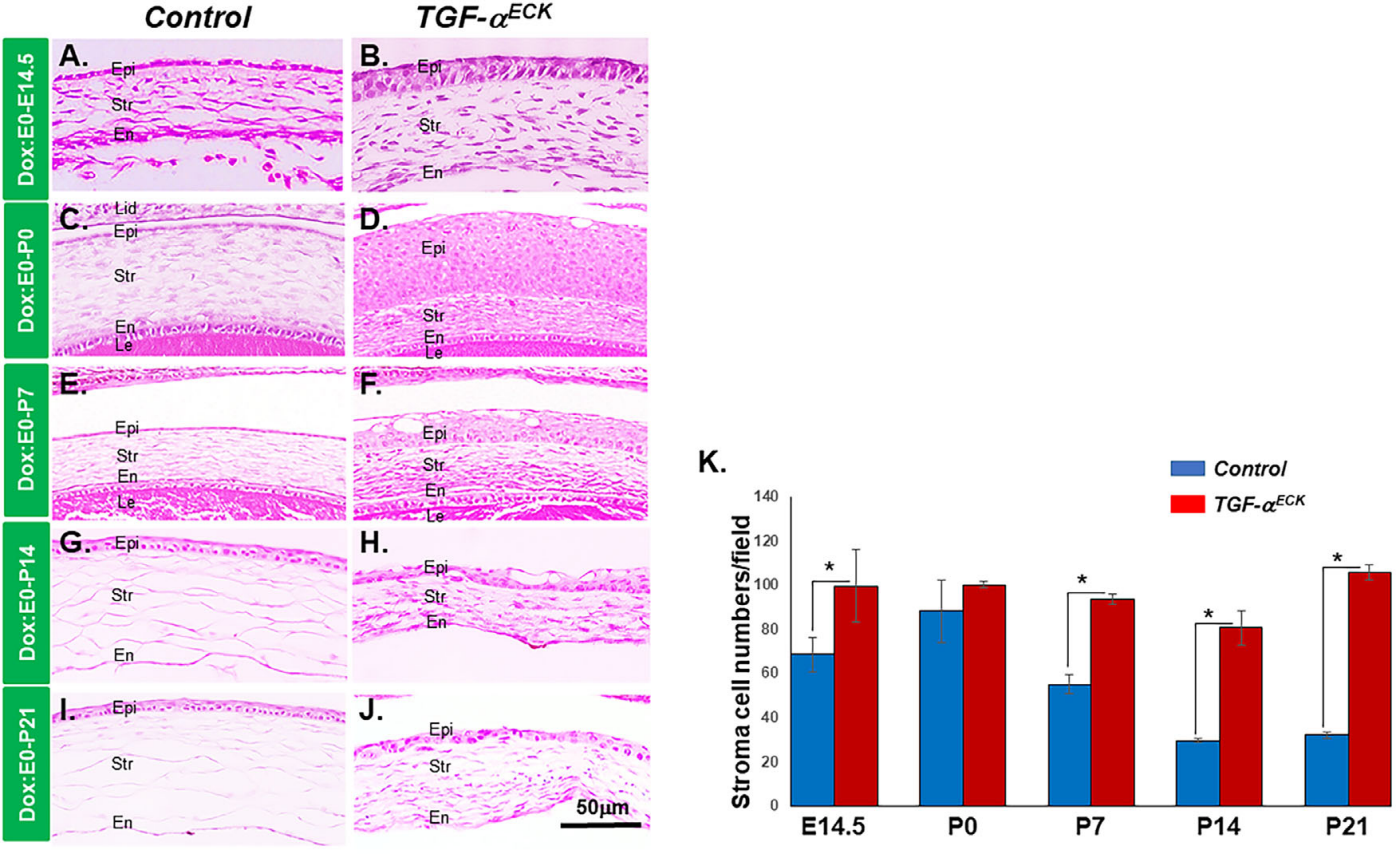
Overexpression of TGF-α in corneal keratocytes resulted in changed phenotypes in corneal epithelium and stroma. (**A**–**J**) Histologic hematoxylin and eosin staining of central cornea sections of *TGF-*α*^ECK^* mice (**B**, **D**, **F**, **H**, **J**) and littermate controls (**A**, **C**, **E**, **G**, **I**) with Dox induction at different stage. Excess TGF-α caused corneal epithelial hyperplasia (**B**, **D**) followed by degeneration (**F**, **H**, **J**). (**K**) Statistical analysis of the corneal stromal cell numbers presented in the central corneas under each microscopic field of view (*n* = 6). The cell density of corneal keratocytes in littermate controls increased at around P0 and then decreased later after that until P14; however, overexpression of TGF-α increased corneal stromal cells at E14.5 and did not decrease after that. En, endothelium; Epi, epithelium; Le, lens; lid, eyelid; str, stroma.

The *TGF-*α*^ECK^* mice treated by Dox administration from E0 till P21 displayed corneal opacities (Figs. 1C, 1D). This phenotype was similar to that observed previously in TGF-α ectopic expression in corneal epithelium.^14^ To further investigate and characterize the phenotypical changes in the cornea of *TGF-*α*^ECK^* mice, Dox induction was performed from E0 until various developmental time points including E14.5, P0, P7, P14, and P21. At each time point, experimental mice were euthanized to collect eyeballs for analysis. Hematoxylin and eosin staining showed that central corneal epithelium in the littermate controls consisted of one or two cell layers at E14.5, P0, and P7 (Figs. 2A, 2C, 2E). Then corneal epithelium had three to four cell layers at P14 (Fig. 2G) and five to six layers at P21 owing to epithelial cell stratification (Fig. 2I). In contrast, TGF-α overexpression resulted in three to four epithelial cell layers at E14.5 (Fig. 2B) and more than 10 cell layers formed by P0 (Fig. 2D). Interestingly, corneal epithelium then gradually showed signs of degeneration and a progressive decrease in cell layers starting at P7 (Figs. 2F, 2H, 2J), which was characterized by the presence of intercellular cavities within the corneal epithelium (Figs. 2D, 2F, 2H) of the *TGF-*α*^ECK^* mice with prolonged Dox induction. In addition, the cell density of corneal stromal cells in littermate controls increased up to P0, and then decreased after that time point until P14; however, overexpression of TGF-α increased corneal stromal cells at E14.5 and remained high after P14 (Fig. 2K).

### Excess TGF-α Altered Cell Phenotype of Corneal Epithelial Cells

**Figure 3.**
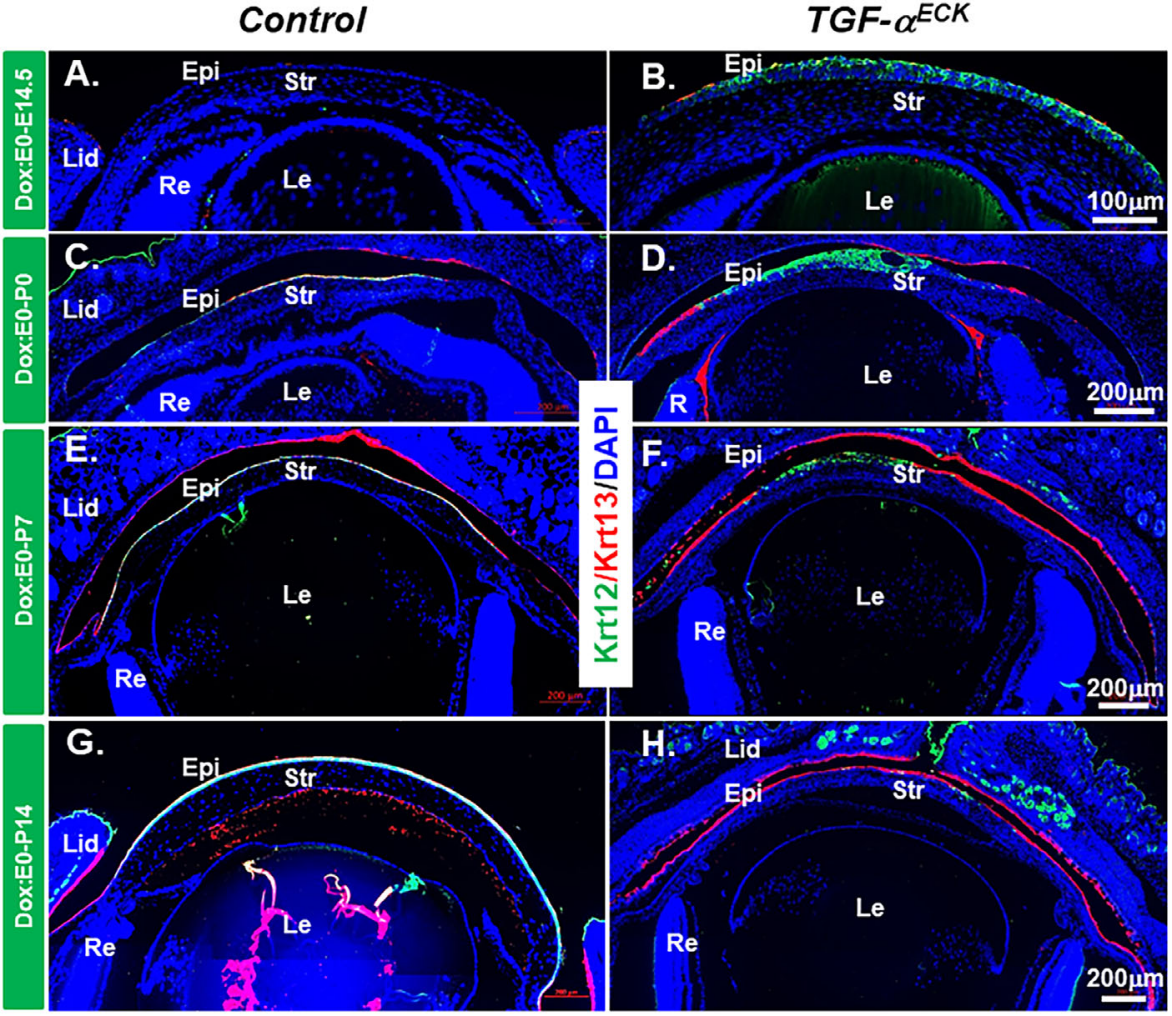
Expression patterns of Krt12 and Krt13 were altered in *TGF-*α*^ECK^*mice. (**A**, **B**) Immunofluorescent staining showed that TGF-α overexpression promoted K12 expression (*green*) in the cornea at E14.5. (**C**–**H**) K12 expression was gradually lost from the peripheral corneal regions at P0 and P7 (**D**, **F**) and almost completely absent at P14 (**H**). By contrast, Krt13 expression (*red*) was progressively extended to the central cornea in *Kera^RT^*/*TGF-*α mice (**D**, **F**) and almost completely covered the cornea at P14 (**H**). Nuclei were counterstained with DAPI (*blue*). En, endothelium; Epi, epithelium; Le, lens; lid, eyelid; Re, retina; str, stroma.

**Figure 4.**
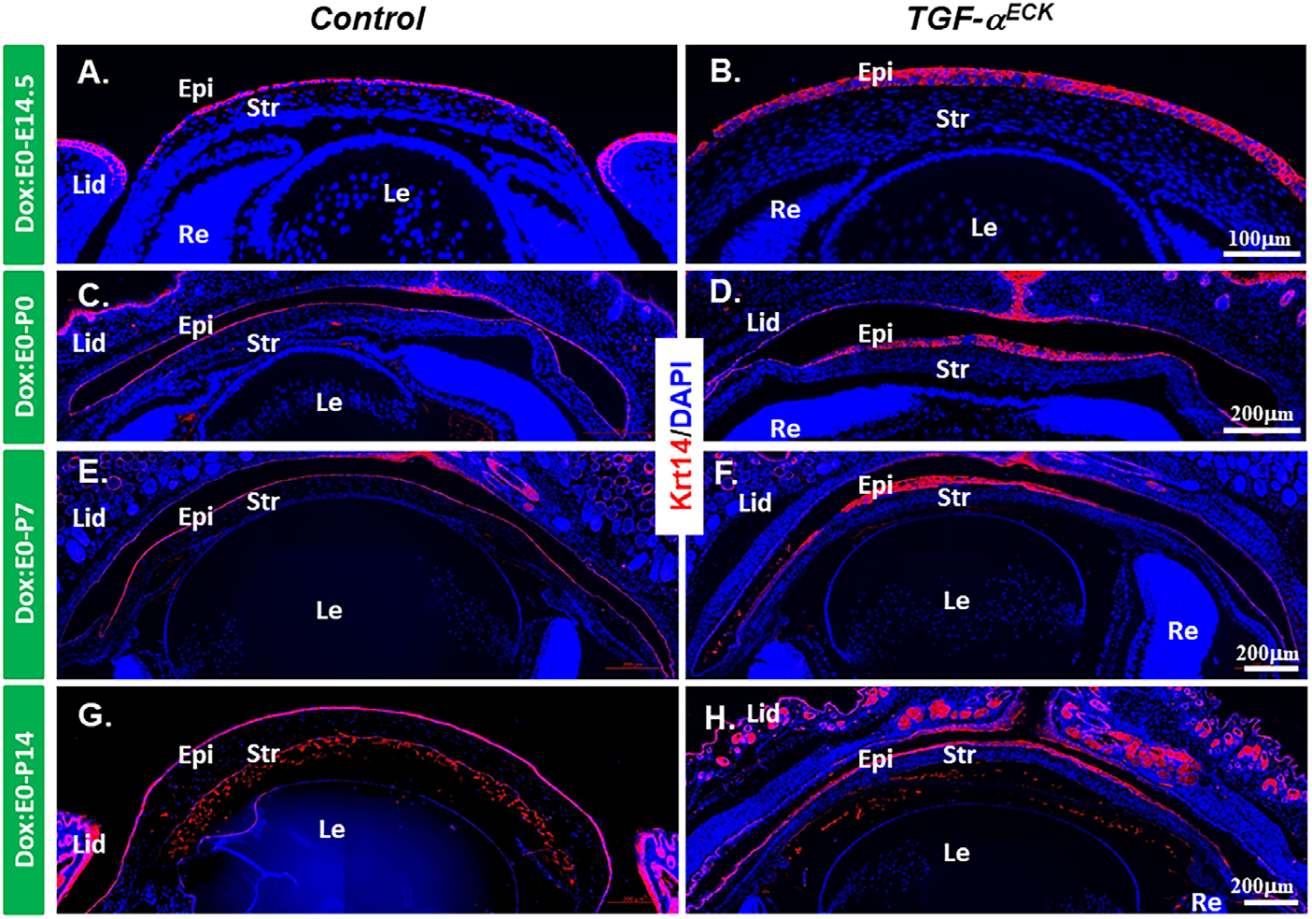
Krt14 expression in corneal epithelium was changed after overexpression of TGF-α. (**A**–**H**) Immunofluorescent-stained of Krt14 (*red*) in the littermate controls and *TGF-*α*^ECK^*mice were administrated Dox from E0 to P7. Krt14 expression existed only in the basal layer of corneal epithelium in the control mice (**A**, **C**, **E**, **G**), but its expression appeared in all the layer of corneal epithelium of *TGF-*α*^ECK^*mice. The nuclei were counterstained with DAPI (*blue*). En, endothelium; Epi, epithelium; Le, lens; lid, eyelid; Re, retina; str, stroma.

Considering TGF-α as a potent mitogen, the EGFR signaling pathway triggered by TGF-α have profound effects on cell differentiation of corneal epithelium and stroma.^15^ To this end, the expression of corneal epithelial differentiation markers was analyzed by immunofluorescent staining. K12, a specific corneal epithelium differentiation marker, was expressed in corneal epithelium after E14.5 in littermate controls (Figs. 3A, 3C, 3E, 3G). Interestingly, K12 expression (green) was detected at E14.5 in the corneal epithelium of *TGF-*α*^ECK^* mice (Fig. 3B), but its expression was gradually diminished from the peripheral corneal region to central corneal epithelium commencing at P0 and thereafter (Figs. 3D, 3F, 3H) and replaced by K13 expressing cells (red), a conjunctival epithelial cell marker, suggesting that persistent expression of TGF-α caused conjunctivalization of *TGF-*α*^ECK^* mouse cornea (Figs. 3D, 3F, 3H). K14 expression is normally restricted to the basal cells of stratified epithelium, including corneal epithelium; however, its expression was found in all the corneal epithelial cell layers of *TGF-*α*^ECK^* mice at various developmental stages, for example, E14.5, P7 and P14 (Fig. 4). Pax6, a transcription factor essential for ocular surface epithelial differentiation, was ubiquitously expressed in the corneal epithelium of both littermate controls and *TGF-*α*^ECK^* mice at all developing stages examined as shown above (Supplementary Fig. S1A–S1H). Taken together, our data indicated that excess TGF-α altered proliferation and differentiation during embryonic corneal morphogenesis.

### Excess TGF-α Altered the Ultrastructure of Corneal Epithelium

**Figure 5.**
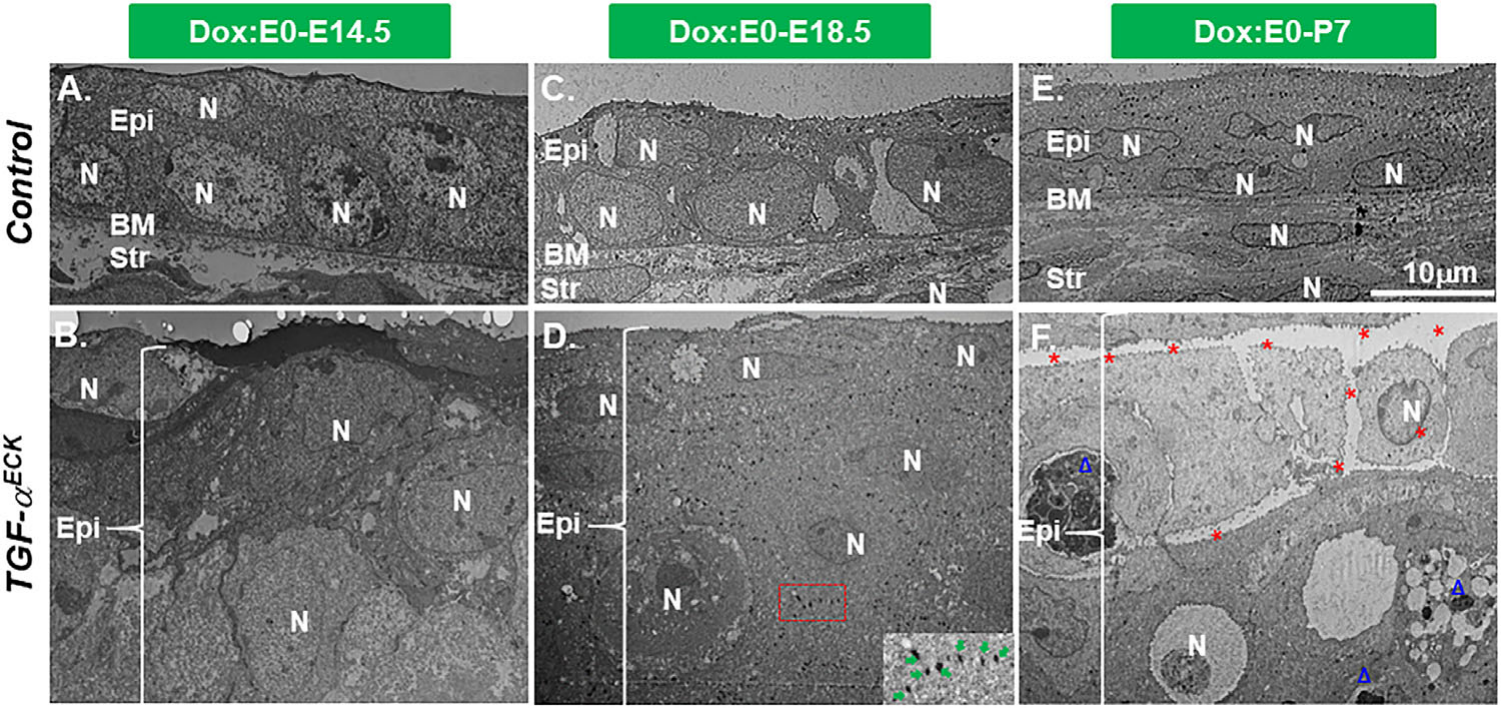
The ultrastructure of corneal epithelium of littermate controls and *TGF-*α*^ECK^* mice at different developmental time points. Corneal epithelium of littermate controls had one to two cell layers at E14.5, E18.5, and P0 (**A**, **C**, **E**). Desmosomes between cells were observed at P7 (**E**). By contrast, corneal epithelium of *TGF-*α*^ECK^*mice had multiple cell layers at all the checked time points (**B**, **D**, **F**). Desmosomes were clearly and densely present in corneal epithelium at E18.5 (**D**). However, corneal epithelial cells lost desmosomes in the superficial layer at P7 (**F**). Note that dark nuclei and condensed fragmented chromatin (*arrowhead*) were evident in some superficial cells. Furthermore, gaps were present between epithelial cells (* in **F**). BM, basal membrane; Epi, epithelium; N, nuclei; str, stroma.

Next, we took a closer look at the ultrastructure of corneal epithelium with transmission electron microscopy. The littermate control mice had one to two corneal epithelial cell layers at E14.5, E18.5, and P7 (Figs. 5A, 5C, 5E). In contrast, there were multiple corneal epithelial cell layers in *TGF-*α*^ECK^* mice at the same developmental stages, respectively (Figs. 5B, 5D, 5F). Furthermore, transmission electron microscopy showed that desmosomes between corneal epithelial cells were first observed in the *TGF-*α*^ECK^* corneal epithelium at E18.5 (Fig. 5D). Although desmosomes were present at P7 in corneal epithelium of littermate controls (Fig. 5E), they were absent in the superficial layers of the *TGF-*α*^ECK^* corneal epithelium at P7 (Fig. 5F). This dynamic change indicated that TGF-α initially promoted desmosome formation in the corneal epithelium, but it caused a loss of desmosomes at later developing stages. Accompanying desmosomes reduction, condensed and fragmented chromatins (arrowhead in Fig. 5F) were observed in some of these superficial cells. Furthermore, gaps were present between cells (asterisks in Fig. 5F). Taken together, these data suggested that excess TGF-α initially caused corneal epithelium hyperplasia, but subsequent progressive degeneration possibly owing to cells undergoing apoptosis.

### Excess TGF-α Caused Phenotypic Changes of Corneal Keratocytes

**Figure 6.**
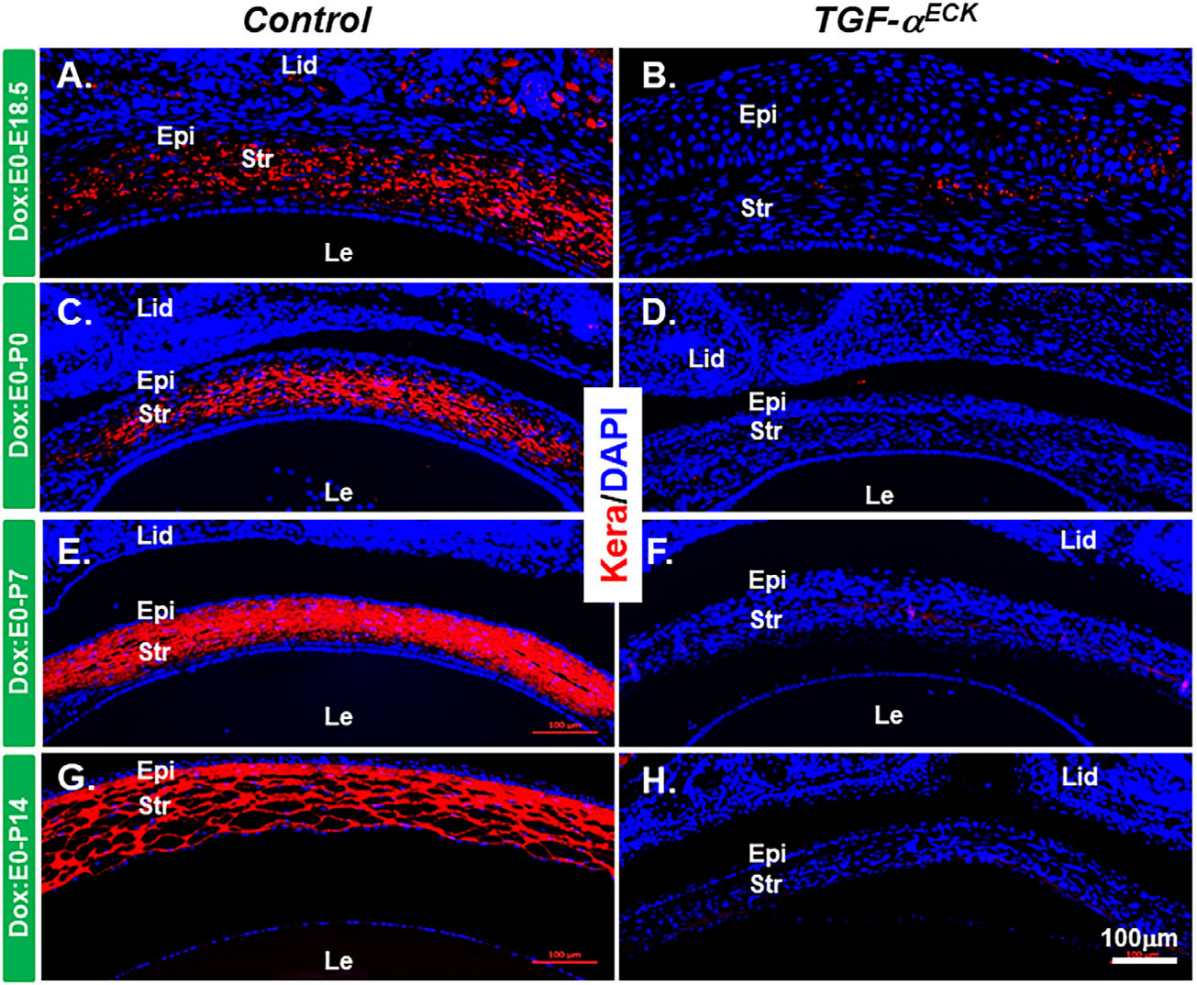
Kera expression was diminished in *TGF-*α*^ECK^* mice. (**A**–**F**) Immunofluorescent-stained micrographs of Kera (*rd*) showed that no positive signals were detected in *Kera^RT^*/*TGF-*α mice after Dox induction. (**B**, **D**, **F**), whereas strong signals were observed in corneal stroma of littermate controls (**A**, **C**, **E**). Cell nuclei were counterstained with DAPI (*blue*). En, endothelium; Epi, epithelium; Le, lens; lid, eyelid; Re, retina; str, stroma**.**

**Figure 7.**
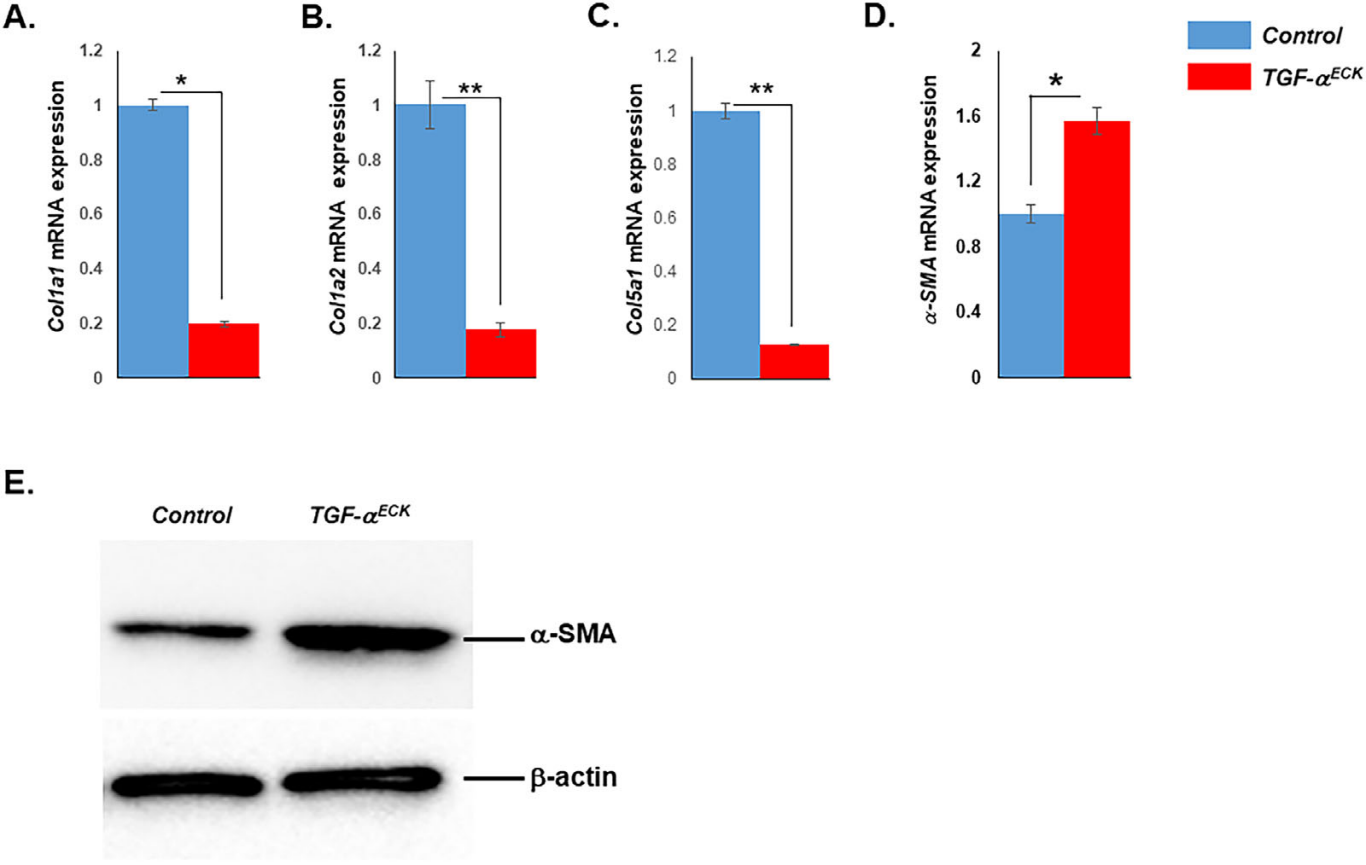
Corneal stromal extracellular matrix components and α-SMA expression were changed in *TGF-*α*^ECK^* mice after Dox induction from E0 to P30. (**A**–**C**) Real-time PCR analysis showed that expression of corneal stroma extracellular matrix components including collagen type 1 a1 (**A**), type 1 a2 (**B**), and type 5 a1 (**C**) decreased in corneal stroma of *TGF-*α*^ECK^*mice administered Dox from E0 to P30. (**D**) Real-time PCR analysis indicated that expression of α-SMA was upregulated in corneal stroma of *TGF-*α*^ECK^*mice administered Dox from P0 to P30. Likewise, (**E**) Western blot results showed that α-SMA protein also increased in the stroma when compared with littermate controls.

The stromal extracellular matrix is critical to corneal function by establishing the appropriate mechanical stability required for maintaining corneal shape and curvature.^16^ To examine the effects of excess TGF-α on the corneal keratocytes, keratocan (Kera) expression was examined by immunofluorescent staining. To our surprise, Kera expression was decreased significantly at E18.5 and totally abolished at P0 (Figs. 6B, 6D, 6F, 6H), whereas strong Kera expression was maintained in the corneal stroma of littermate controls (Figs. 6A, 6C, 6E, 6G). Moreover, RT-qPCR data showed that expression of *Col1a1*, *Col1a2*, and *Col5a1* dramatically decreased in *TGF-*α*^ECK^* stroma when compared with that in littermate controls (Figs. 7A, 7B, 7C). However, the expression of alpha-smooth muscle actin (α-SMA) was significantly increased in Dox-induced *TGF-*α*^ECK^*stroma, as revealed by RT-qPCR and Western blotting (Figs. 7D, 7E). These data suggested that changes of keratan sulfate proteoglycans and extracellular matrix assembly might have some influence on the corneal transparency and lead to corneal haze as observed in *TGF-*α*^ECK^*mice.

### TGF-α Promoted Corneal Epithelium and Stromal Cell Proliferation

**Figure 8.**
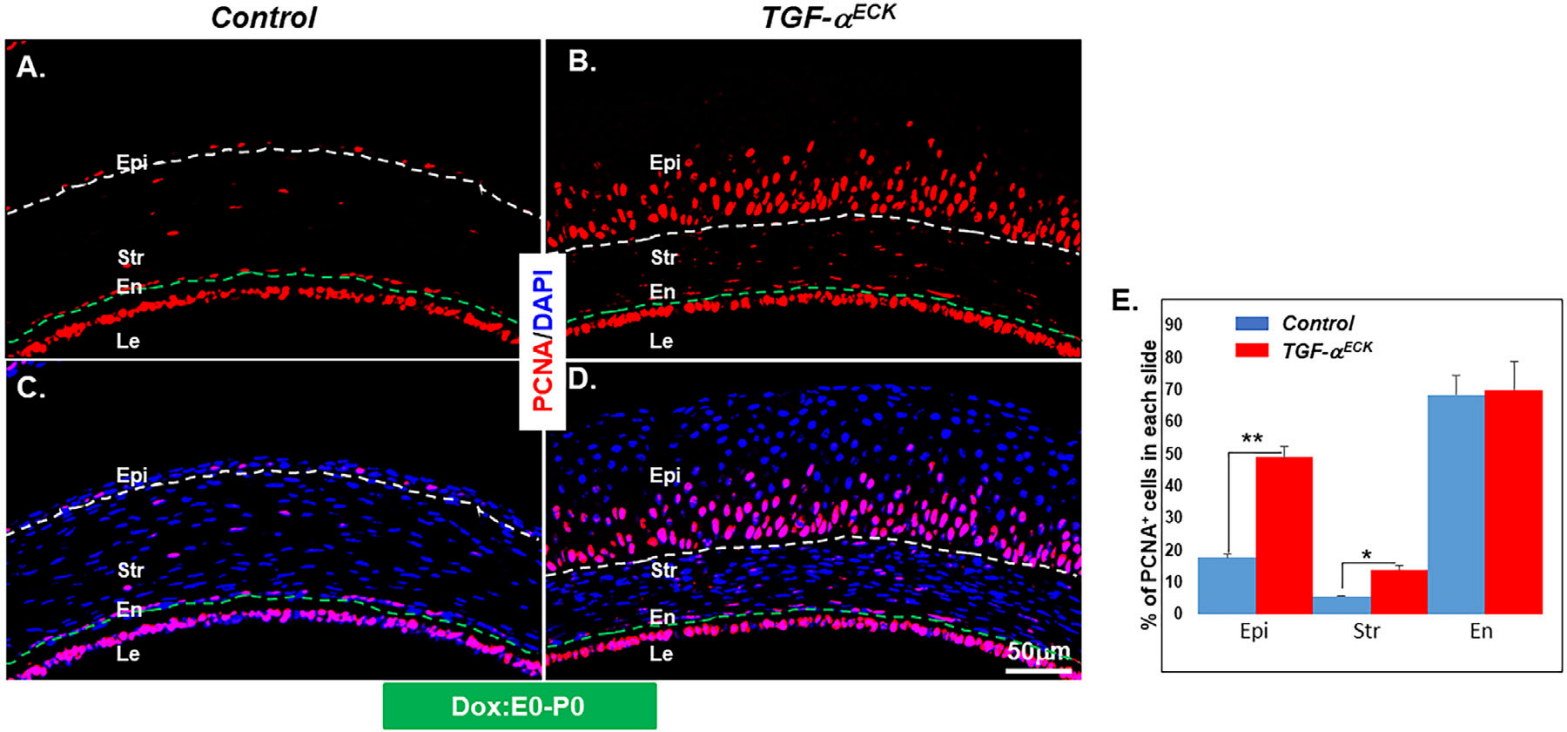
TGF-α overexpression increased cell proliferation in corneal epithelium and stromal. (**A**–**D**) Immunofluorescent micrographs of the corneas probed with anti-PCNA showing a few positive PCNA (PCNA**^+^**) cells scattered at the basal corneal epithelial cells of littermate controls. In contrast, abundant PCNA**^+^** corneal epithelial cells were present in *TGF-*α*^ECK^*mice administrated Dox from E0 to P0 (**A** vs **B**, **C** vs **D**). Similarly, PCNA**^+^** stromal cells were enhanced in *Kera^RT^*/*TGF-*α mice when compared with the littermate controls. The *white and green dashed lines* in each panel delineate the corneal epithelial layer and endothelial cells, respectively. Nuclei were counterstained with DAPI (*blue*). (**E**) Statistical analysis of immunofluorescent staining results showed that the percentage of PCNA positive cells in epithelium, stroma, and endothelium of littermate controls and *TGF-*α*^ECK^*mice (*n* = 4). En, endothelium; Epi, epithelium; Le, lens; str, stroma.

Multiple cell layers in the corneal epithelium and higher stromal cell density implicated that cell proliferation was upregulated in the Dox-induced *TGF-*α*^ECK^*mice. To address this hypothesis, the expression of proliferating cell nuclear antigen (PCNA) was examined by immunofluorescent staining in mice at P0 (Dox induction beginning at E0). It showed that few of PCNA^+^ cells were detected in corneal epithelium, stroma, and endothelium of littermate controls at P0 (Figs. 8A, 8C). However, PCNA^+^ cells were plentifully present in the *TGF-*α*^ECK^*corneal epithelium and stroma (Figs. 8B, 8D). PCNA^+^ cells in *TGF-*α*^ECK^*endothelium showed no change in comparison with controls (Fig. 8B vs Figs. 8A, 8D vs Fig. 8C). Statistical analysis of PCNA immunofluorescent immunostaining data indicated that cell proliferation was increased in corneal epithelial and stromal cells of *TGF-*α*^ECK^*mice at this developmental stage. However, endothelial cell proliferation did not change in Dox-induced *TGF-*α*^ECK^*mice (Fig. 8E). These data indicated that TGF-α was a potent stimulator of corneal epithelial cells and stromal cells during mouse development.

## Discussion

In the present study, we found that excess TGF-α produced from corneal stromal cells had a profound influence on corneal development in the *TGF-*α*^ECK^*mice. Our data indicated that TGF-α initially promoted corneal epithelial cell hyperplasia, followed by degeneration; which was accompanied by the loss of K12 expression, whereas ectopic expression of K13 resembling conjunctivalization of corneal epithelium after prolonged Dox induction, a phenotype characteristic of limbal stem cells deficiency.^26^ Interestingly, excess TGF-α caused phenotypic changes of keratocyte, such as loss of Kera expression and downregulation of extracellular matrix components, and upregulation of α-SMA, a maker of myofibroblasts. All these defects during corneal development resulted in corneal opacities in *TGF-*α*^ECK^*mice. Therefore, overexpression of TGF-α in keratocytes was detrimental to normal corneal morphogenesis.

The overexpression of TGF-α specifically in corneal stroma of *TGF-*α*^ECK^ mice* initially increased corneal cell proliferation both in the stroma and the epithelium. This phenomenon was expected because TGF-α is a potent mitogen for epithelial cells and fibroblasts and can affect cell behavior in an autocrine, paracrine, or even juxtacrine manner.^5^^,^^6^^,^^17^ However, we noticed that corneal endothelium in the *TGF-*α*^ECK^*mice were not perturbed by excess TGF-α. This finding is not consistent with previous observations; Reneker et al.^10^^,^^11^ reported that the overexpression of TGF-α in the ocular lens via the mouse αA-crystallin promoter caused no endothelium formation and multiple anterior segment defects in the transgenic mice. The αA-*crystallin* gene was expressed in mouse lens starting at E12.5 before the formation of mouse corneal endothelium at E14.5 to E15.5,^18^ *whereas* *Kera* gene expression in the corneal stroma commences at E14.5.^19^ Therefore, different mouse drivers activated TGF-α overexpression at different developmental stages may account for the variegation of phenotypes observed in these mouse strains.

One interesting observation in the present studies was that excess TGF-α driven by *TGF-*α*^ECK^* led to corneal epithelial hyperplasia that was followed by subsequent epithelium degeneration (Fig. 2), likely via apoptosis (shown in Fig. 5), which was accompanied by the conjunctivalization of cornea epithelium (Fig. 3). This observation was unique and distinct from phenotypic changes found in other transgenic mouse lines that overexpress TGF-α and fibroblast growth factor-7 in eyelid stroma and corneal epithelial cells, respectively.^20^^,^^21^ It has been reported that overexpression of EGF and/or EGFR caused apoptosis of cultured epithelial cells in vitro.^22^^,^^23^ Thus, it is likely that excess TGF-α promoted corneal epithelial cell proliferation during embryonic development. However, it might lead to apoptosis at postnatal corneal epithelium under pathologic conditions owing to dysregulation of the cell cycle.^24^^,^^25^ The conjunctivalization could be in part explained by the rapid turnover rate of corneal epithelial cells under the influence of excess TGF-α, which might exhaust the limbal stem cells resembling the phenomenon observed in limbal deficiency.^26^^,^^27^ This idea will be investigated using our new driver moue strain, *Krt4-rtTA* in a future study in which the *rtTA* minigene was knocked into the mouse *Krt4* locus (Zhang J, et al. *IOVS* 2016;57:ARVO E-Abstract 12).

Another interesting finding in this study was that Kera expression diminished at P0 in the corneal stroma of *TGF-*α*^ECK^*mice with Dox induction from E0 (Fig. 6). This finding implied that de novo rtTA synthesis might no longer take place in the stroma of *TGF-*α*^ECK^* mice at P0; however, rtTA protein might persist and maintain TGF-α expression, alternatively, which would lead to the termination of *TGF-*α overexpression, but the influence of excess TGF-α from E0 through P0 might reach a point of no return and irreversibly alter the cell fate of corneal epithelial cells and stromal cells of *TGF-*α*^ECK^*mice.

Kera is one of the important keratan sulfate proteoglycans uniquely expressed in the corneal stroma and plays a crucial role in the development and maintenance of corneal transparency^19^; therefore, the loss of Kera expression affects corneal transparency. Extracellular matrix assembly have also been implicated in corneal transparency.^28^^–^^31^ Collagen I and collagen V are two fibril-forming collagens synthesized by keratocytes,^30^^,^^32^ and our data indicated that both types of collagen decreased significantly (Figs. 7A–7C), which might contribute to the corneal haze. In contrast, our RT-qPCR and Western blot results showed that α-SMA expression was boosted in corneal stroma of *TGF-*α*^ECK^*mice (Figs. 7D, 7E). Upregulation of α-SMA may be triggered by inflammatory cytokines released from corneal inflammatory cells owing to excess TGF-α in the cornea. In the double transgenic mouse strain, *K12_rtTA_/TGF-*α, corneal inflammation was reported after TGF-α overexpression in epithelial cells (Kao WW-Y, et al. *IOVS* 2011;52: ARVO E-Abstract 309). In the same mouse model, increased expression of Wnt5a was also found after excess TGF-α was produced in the cornea.^14^ Wnt5a is an inflammatory mediator that promotes inflammatory response.^33^ Thus, in our current *TGF-*α*^ECK^*mice, TGF-α overexpression may indirectly result in corneal inflammation, which leads to the expression of α-SMA. α-SMA was known as one of the hallmarks of myofibroblast, and the presence of α-SMA may imply that some corneal stromal cells might undergo transformation. The exact mechanism of this transformation caused by TGF-α will be investigated in future studies.

In our current study, we used the novel knock-in driver mouse line *Kera^RT^* to express TGF-α in corneal keratocytes, *Kera^RT^* mouse line displayed strong rtTA expression in Kera expressing cells.^13^ Previously, we generated a transgenic mouse line called KR by randomly inserting the *Kera_promoter_-rtTA* minigene into the mouse genome, in which expression of rtTA was driven by mouse Kera promoter region.^34^ The double transgenic mouse *KR/TGF-*α was created to express TGF-α in corneal stromal cells with Dox administration. The eyes of Dox-induced *KR/TGF-*α mice looked normal and transparent^20^; no dramatic phenotypes were observed. This inconsistent consequence of ectopic expression of TGF-α in cornea of these two mouse strains is probably owing to different expression levels of TGF-α in their corneal stroma, which suggested that lower TGF-α level was produced in *KR/TGF-*α mouse strain. Reneker et al.^16^^,^^17^ also reported that different level of TGF-α expression caused variable phenotypes in their transgenic mouse lines. Based on our experiences, *Kera^RT^* knock-in mouse line (driver gene activity mainly in the corneal stroma) was more effective and faithful in driving transgene expression mimicking Kera expression patterns limited in corneal stroma of *TGF-*α*^ECK^*mice, whereas transgenic mouse line *KR* (driver gene activity mainly in eyelid stroma) is to modify gene expression in mouse eyelid stroma.

## Conclusions

Results of our studies indicated that excess TGF-α synthesized by corneal stromal cells led to conjunctivalization of corneal epithelium expressing K13 resembling limbal stem cells deficiency and transition of keratocytes to assume myofibroblastic cell fate, for example, enhanced α-SMA expression.

## Supplementary Material

Supplement 1

Supplement 2
