# Supplementary material for: Excess Transforming Growth Factor-α Changed the Cell Properties of Corneal Epithelium and Stroma
Source: Invest Ophthalmol Vis Sci. 2020 Jul 15;61(8):20. doi: 10.1167/iovs.61.8.20 (PMC7425719; doi:10.1167/iovs.61.8.20)
Supplement: Supplement 1 [file iovs-61-8-20_s001.pdf]

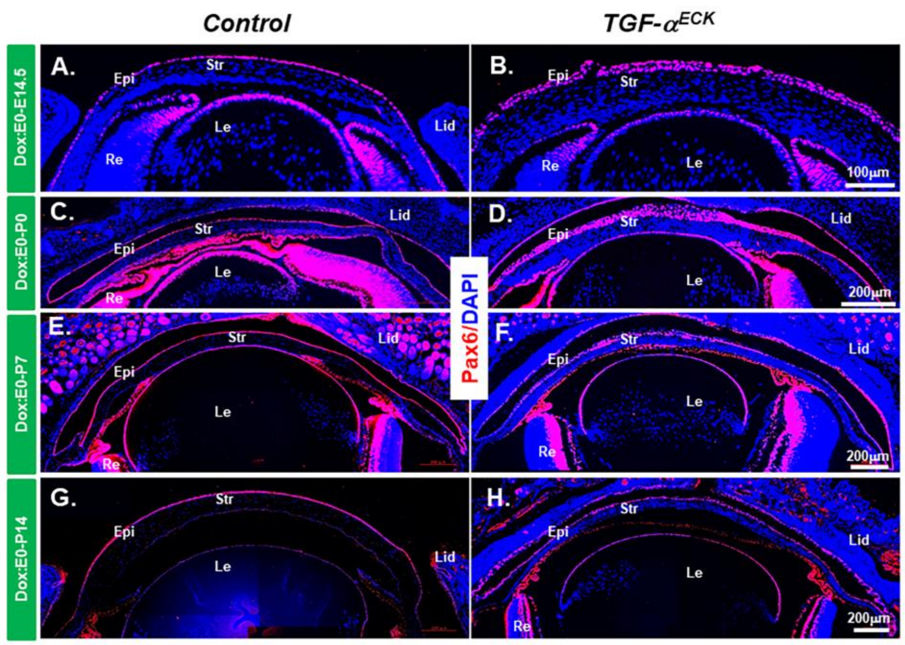

Supplementary Figure 1. Pax6 expression did not change in Dox-induced *TGF-*α^{ECK} mice. (A-H) Immunofluorescent micrographs showed that just like the littermate controls, the corneal epithelial cells were Pax6 positive (red), regardless of Dox induction stage. (A, B) Dox: E0-E14.5; (C, D) Dox: E0-P0; (E, F) Dox: E0-P7; (G, H) Dox: E0-P14. Cell nuclei were counterstained with DAPI (blue). En, endothelium; Epi, epithelium; Le, lens; str, stroma.

Sup. 2. Zhang

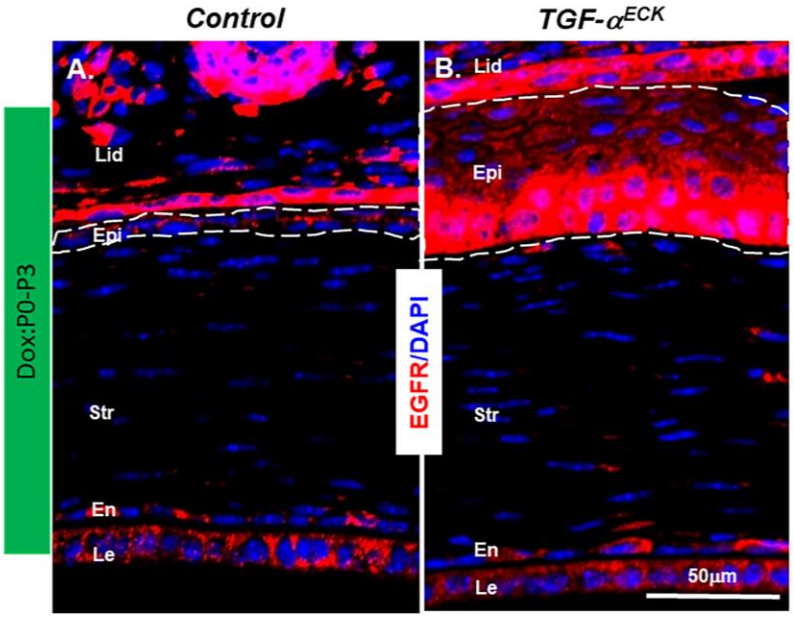

Supplementary Figure 2. EGFR expression was increased in Dox-induced *TGF-α^ECK* mice. (A-B) Immunofluorescent micrographs showed that EGFR expression in the corneal epithelium and stromal cells was increased after Dox-induction from P0-P3 (red). Cell nuclei were counterstained with DAPI (blue). Lid, eyelid; En, endothelium; Epi, epithelium; Le, lens; str, stroma.
