# Supplementary material for: Excess Transforming Growth Factor-α Changed the Cell Properties of Corneal Epithelium and Stroma
Source: Invest Ophthalmol Vis Sci. 2020 Jul 15;61(8):20. doi: 10.1167/iovs.61.8.20 (PMC7425719; doi:10.1167/iovs.61.8.20)
Supplement: Supplement 2 [file iovs-61-8-20_s002.pdf]

Supplementary Table 1: PCR Primers used for genotyping or RT-qPCR in this study

Transgenic mouse line/Genes	Sequence	Application
$Kera^{RT}$	5' –TGGTGGCTTGCTTCAAGCTTCTTC-3'<br>5' –TATCCAACTCACAACGTGGCACTG-3'<br>5' –GGAGTCTGCACTACCAGTACTCAT-3'	For genotyping<br>Knock-in: 462bp; WT:389bp
tetO-TGF-a	5' –CCTGTTCGCTCTGGGTATTGTGTT-3'<br>5' –CGTGGTCCGCTGATTTCTTCTCTA-3'	For genotyping<br>transgenic line: ~300bp; WT, no band
Col1a1	5' –GAGCTCAGAGGCGAAGGCAACA<br>5' –GCCAATGTCTAGTCCGAATTCC	For RT-qPCR
Col1a2	5' –GTTCACCTACTCTGTCCTAGTC<br>5' –TGTCCAGAGGTGCAATGTCAAG	For RT-qPCR
Col5a1	5' –TTCCTCAGCCGCAGTGACCA<br>5' –GGCAGCTCGGTTGTCAGAGA	For RT-qPCR
Aldh3a1	5' – GACATCAAGCGGTGGAGTGACA<br>5' –GCAGAGACCTCACCAGCAAGA	For RT-qPCR
α-SMA	5' – GGATGGAGTCAGCGGGCATC<br>5' – TGGAGCCACCGATCCAGACA	For RT-qPCR
Gapdh	5'– AAGGTGGTGAAGCAGGCATCTGAG<br>5' –TCTTACTCCTTGGAGGCCATGTAG	For RT-qPCR

Supplementary Table 2: Antibodies used for IHC or WB in this study

Primary/Secondary antibodies	Host	Source	Application
anti–Krt12	Rabbit	Customer made	IHC (1:200)
anti–keratocan	Rabbit	Customer made	IHC (1:200)
anti–Krt13	Mouse	Cat#ab16112; Abcam	IHC (1:200)
anti-Krt14	Mouse	Cat#ab7800; Abcam	IHC (1:200)
anti-p63(63P02)	Mouse	Cat#MS-1082-P; Neomarkers	IHC (1:200)
anti-Pax6	Rabbit	Cat#PRB-278P; Biolegend	IHC (1:200)
anti-EGFR	Rabbit	Cat#06-847; Millipore	IHC (1:200)
anti-PCNA(PC10)	Rabbit	Cat#ab2426; Abcam	IHC (1:200)
anti-TGF-a	Rabbit	Cat#ab9585; Abcam	WB(1:1000)
anti-a-SMA	Rabbit	Cat#ab5694; Abcam	WB(1:1000)
anti-b-Actin	Mouse	Cat#A2228; Sigma-Aldrich	WB (1:1000)
Anti-Rabbit IgG-Alexa-488	Goat	Molecular Probes, Inc	IHC (1:200)
Anti-Rabbit IgG-Alexa-594	Goat	Molecular Probes, Inc	IHC (1:200)
Anti-Mouse IgG-Alexa-594	Donkey	Molecular Probes, Inc	IHC (1:200)
anti Rabbit IgG- HRP Conjugate	Goat	Cat#1706515; Bio-Rad Laboratories, Inc.	WB (1:3000)
anti Mouse IgG-HRP Conjugate	Goat	Cat#1721011; Bio-Rad Laboratories, Inc.	WB (1:1000)

WB-Western Blot; IHC-Immunohistofluoscent staining
